# Pathogenic mechanisms and therapeutic promise of phytochemicals and nanocarriers based drug delivery against radiotherapy-induced neurotoxic manifestations

**DOI:** 10.1080/10717544.2022.2064562

**Published:** 2022-05-11

**Authors:** Ashif Iqubal, Mohammad Kashif Iqubal, Sumit Sharma, Mohd Wasim, Mohamed A. Alfaleh, Shadab Md, Sanjula Baboota, Javed Ali, Syed Ehtaishamul Haque

**Affiliations:** a Department of Pharmacology, School of Pharmaceutical Education and Research, Jamia Hamdard, New Delhi, India; b Department of Pharmaceutics, School of Pharmaceutical Education and Research, Jamia Hamdard, New Delhi, India; c Product Development Department, Sentiss Research Centre, Sentiss Pharma Pvt Ltd, Gurugram, India; d Department of Pharmaceutics, Faculty of Pharmacy, King Abdulaziz University, Jeddah, Saudi Arabia; e Vaccines and Immunotherapy Unit, King Fahd Medical Research Center, King Abdulaziz University, Jeddah, Saudi Arabia; f Center of Excellence for Drug Research & Pharmaceutical Industries, King Abdulaziz University, Jeddah, Saudi Arabia

**Keywords:** Cognitive dysfunction, hippocampus, chemotherapy, NF-kB and miRNA

## Abstract

Radiotherapy is one of the extensively used therapeutic modalities in glioblastoma and other types of cancers. Radiotherapy is either used as a first-line approach or combined with pharmacotherapy or surgery to manage and treat cancer. Although the use of radiotherapy significantly increased the survival time of patients, but its use has been reported with marked neuroinflammation and cognitive dysfunction that eventually reduced the quality of life of patients. Based on the preclinical and clinical investigations, the profound role of increased oxidative stress, nuclear translocation of NF-kB, production of proinflammatory cytokines such as TNF-α, IL-6, IL-β, increased level of MMPs, increased apoptosis, reduced angiogenesis, neurogenesis, and histological aberrations in CA1, CA2, CA3 and DG region of the hippocampus have been reported. Various pharmacotherapeutic drugs are being used as an adjuvant to counteract this neurotoxic manifestation. Still, most of these drugs suffer from systemic adverse effect, causes interference to ongoing chemotherapy, and exhibit pharmacokinetic limitations in crossing the blood-brain barrier. Therefore, various phytoconstituents, their nano carrier-based drug delivery systems and miRNAs have been explored to overcome the aforementioned limitations. The present review is focused on the mechanism and evidence of radiotherapy-induced neuroinflammation and cognitive dysfunction, pathological and molecular changes in the brain homeostasis, available adjuvants, their limitations. Additionally, the potential role and mechanism of neuroprotection of various nanocarrier based natural products and miRNAs have been discussed.

## 1. Introduction

Brain tumor (BT) is one of the commonly diagnosed solid tumor among children and adults in the United States. Although BT is less widely diagnosed than other types of cancer and accounts for 1% of total confirmed cases (Lee et al., 2012). It was forecasted that by the end of 2021, 83,570 new cases would be diagnosed and out of which 29.7% of malignant BT and 70.3% of nonmalignant BT cases will be reported (Miller et al., 2021). It was further reported that the survival rate of BT patients is comparatively low, and only 36% of total confirmed cases survive for more than 5 years (Miller et al., 2021). Until now, the exact cause and mechanism of BT is not confirmed but based on the preclinical and clinical evidence, it was found that genetic factors, environmental toxins, epigenetic modification and exposure to electromagnetic filed are primary causes (Nieblas-Bedolla et al., 2021). Currently, pharmacotherapy, surgery, and radiotherapy are used for the management and treatment, but most of the pharmacotherapeutic drugs suffers from pharmacokinetic and pharmacodynamic limitation. Although Surgical intervention are initially used and it has been reported that once, BT is diagnosed and confirmed, removal of maximum portion of tumor is recommended and than pharmacotherapeutic drugs and radiotherapy is used (Nieblas-Bedolla et al., 2021). The initial surgical procedure significantly reduced the tumor size and volume and hence, reduced the dose of chemotherapeutic drugs and radiotherapy (Nieblas-Bedolla et al., 2021). However, surgical procedure is only recommended for the superficial tumor and for the deep-seated tumor or tumor located near the sensitive area of the brain, this process is not feasible. After successful surgery, chemotherapy drugs, either alone or in combination, is used and many studies have shown synergistic effects and prolonged survival when surgery is combined with pharmacotherapy (Ho & Stea, 2022). However, most conventional and targeted chemotherapeutic exhibit side effects such as nonspecific damage to brain vells, immune suppression, secondary infection, alopecia, hepatotoxicity, cardiotoxicity, bruises etc (Ho & Stea, 2022). Moreover, the blood-brain barrier (BBB)’s presence also impairs the reach of these drugs into the tumor microenvironment.

Another important approach for the treatment of BT is radiotherapy. The radiotherapeutic approach consist of exposure of high-energy X rays and y rays to kill the cancerous cells (Lupattelli et al., 2020). Radiotherapy can either be external or internal (Ho & Stea, 2022). External radiotherapy consists of stereotactic procedure, gamma knife linear accelerator (LINAC) and whole brain radiation (Ho & Stea, 2022). Various clinical studies have shown the benefits of radiotherapy when combined with surgery or chemotherapy, and a significant increase in the survival rate of patients has been reported (Lupattelli et al., 2020). Recent advancements in radiological techniques such as CT scans, MRI, 3 D scans, 3 D-CRT, etc., have tremendously increased the clinical outcomes of radiotherapy (Flores-Castro & Sebastian-Barajas, 2021; Li et al., 2021).

**Figure 1.**
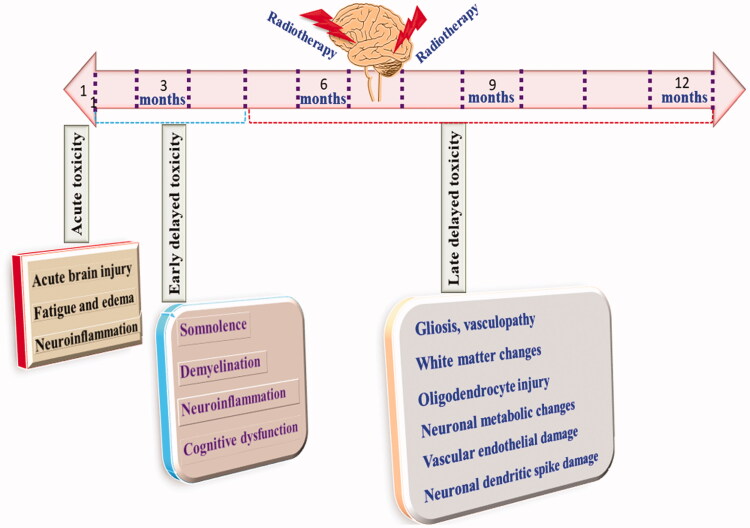
Manifestations and time course of radiotherapy-induced neurotoxic manifestations.

Despite being an important therapeutic modality, radiotherapy has its own serious limitation (Kłos et al., 2019). Numerous reports have shown significant brain injury followed by radiotherapy. Acute, early delayed, and late delayed are brain injury types related to radiotherapy (Pazzaglia et al., 2020). Acute injuries are manifested within 48 h to 7 b weeks are characterized by fatigues, loss of hair, severe vomiting and diarrhea (Pazzaglia et al., 2020). Early delayed rain injury is observed within 6 months of exposure to radiotherapy (Warrington et al., 2013). Clinically symptoms consist of dizziness, somnolescence, demyelination, short-term dementia and behavioral deficit. Late, delayed brain injury is observed after 6 months to years and is typically characterized by vascular dementia, cognitive dysfunction and neuronal necrosis, as shown in Figure 1 (Warrington et al., 2013).

Studies have shown that more than 40–50% of patients after radiotherapy suffers from cognitive dysfunction (Johannesen et al., 2003). Clinical trials have also shown that when WBRT is combined with the stereotactic procedure, severe brain injury and reduced learning and memory functions have been found (Liu et al., 2010). Apart from the clinical evidence, preclinical studies had shown significant memory dysfunction in rats when radiotherapy was given post-surgery. Similarly, after cranial irradiation for 12 months, marked cognitive dysfunction was observed (Warrington et al., 2012). Exposure to gamma radiation for 6–9 months also showed a decline in the learning and memory of rodents (Warrington et al., 2013).

Apart from cognitive dysfunction, WBRT was also reported to cause damage to the pituitary gland, hormonal disbalance, motor dysfunction, and gastrointestinal abnormalities, as shown in Table 1. Until now, the exact pathogenesis of radiotherapy that induces brain damage is not explored, but various studies have shown the role of neuroinflammation, neuronal apoptosis, neurogenesis, demyelination, histopathological damage and alteration in neurochemical status as primary pathogenic factors (Zhang et al., 2018). Additionally, there is no specific treatment available to mitigate the radiation-induced brain damage, and currently used drugs to reduce this issue suffer from their own pharmacokinetic and pharmacodynamic limitations (Zhang et al., 2018). Thus, in the present review, we have discussed evidence-based radiation-induced brain injury, its pathogenic mechanic mechanism, available treatment regimen, and the emerging role of nanocarrier-based therapy to take care of this situation.

**Table 1.** The deleterious effect of radiotherapy in cognitive and hormonal dysfunction (1).

Injury type	Observation	Radiotherapy dose
Cognitive dysfunction	Cognitive dysfunction	25 Gy/single dose 10, 20 and 40 Gy/ single dose 20 Gy/4 dose and 40 Gy/8 dose
	Behavioral dysfunction and depression	6 Gy/single cycle 36 Gy/8 cycle
	Impaired auditory function, attention deficit syndrome, visual and verbal impairment	30–50 Gy
Motor dysfunction	Impairment un locomotion and grip strength	6 Gy/single cycle
Growth hormone alteration	Confirmed by arginine test and insulin tolerance test	50–60 Gy
Cellular and histological changes	Disruption of BBB	18 Gy
	Increased activation and proliferation of microglia	10 Gy
	Astrocytic hypertrophy	8 Gy
	Histological damage	15 Gy
	Enhanced vacuolation, damage to endoplasmic reticulum and mitochondria	2 *0.75 Gy
	Necrosis in hippocampus	2*10, 3*10 and 4*10
	Reduced maturation of neurons	2.5* (whole-body)
	Reduced density of endothelial cells and damage to BBB	0–50 Gy
	Gliosis, necrosis, and damaged BBB	50, 75, and 120 Gy
	Astrogliosis, BBB damage and astrogliosis	20–40 Gy
	Astrogliosis and cognitive dysfunction	40 Gy

## 2. A Glimpse of previously published literature on radiotherapy-induced neurotoxic manifestation

Before proceeding further, it is important to provide short and concise information about the various reviews published on radiotherapy-indued neurotoxic manifestation and novelty of this scholarly work.

No doubt radiotherapy offers cutting edge therapeutic benefit in the treatment of brain tumors, head and neck malignancy, and against other types of tumors (Makale et al., 2017). As most of pharmacotherapeutics have limitation in crossing BBB, and hence, desired therapeutic concentrations is not achieved in the brain, leading to por clinical outcome and reduced quality of life. Thus, either whole brain radiation or whole-body radiation has overcome such limitations (Robbins et al., 2012; Pazzaglia et al., 2020). Despite the descent therapeutic role, radiotherapy has been reported to exhibit various neurotoxic manifestations. Cognitive dysfunction is among the most prevalent neurotoxic attributes. Radiotherapy-induced cognitive dysfunction is associated with reduced learning, memory, depressive-like behavior, anxiety, and dementia (Greene-Schloesser & Robbins, 2012; Turnquist et al., 2020). The mechanistic role of this situation involves damaged BBB, structural damage to neurons, oligodendrocytes, epithelial cells, leucitic infiltration, and discrete release of various neurotransmitters. It was further found that sub granular zone (SGZ), sub ventricular zone (SVZ), and dentate gyrus (DG) are mostly impacted by radiotherapy and lead to neurobehavioral dysfunction (Zhang et al., 2015). Moreover, the exact pathophysiology is not yet clear and it was found that a single cell type is not involved rather, multiple cells and dynamics are functionally involved in the pathogenesis (Kim et al., 2008; Makale et al., 2017).

Furthermore, the molecular and cellular analysis showed that radiotherapy causes microglial activation, regulates the microglial polarization, modulates the leucocytic infiltration, disrupts the BBB, and causes neurotoxic manifestation (Li et al., 2003). Number of published evidence have shown that radiotherapy increases the phosphorylation and nuclear translocation of NF-kB, leading to increased transcription of proinflammatory cytokines such as TNF-α, IL-6, IL-1β etc., and causes neuroinflammation, as well as dementia (Yang et al., 2020). No doubt, BBB acts as a protective barrier against various noxious stimuli, and radiotherapy is well-established to cause breakage of BBB. Studies have shown that exposure to various intensities of X-ray or gamma-ray causes alteration in the level of MMPs, collagens, TIMPs, MAPKs, ERK1/2, resulting in the damage of BBB (Ballesteros-Zebadúa et al., 2012; Constanzo et al., 2020).

Normal neurogenesis is an important factor that regulates the optimum level of neurons in the various region of the hippocampus, such as CA1, CA2, CA3, DG, SGZ, and SVZ. Based on the mechanistic study, it was found that exposure to radiotherapy causes increased production of ROS via modulation of NADPH oxidase and Nrf2, leading to reduced activity of SOD, CAT, GSH, and increased level of MDA (Collins-Underwood et al., 2008; Veeraraghavan et al., 2011).

Nevertheless, increased ROS causes increased expression of proinflammatory chemokines, cytokines, adhesion molecules, and other critical regulators neurotoxic manifestations (Iqubal et al., 2020; Khan et al., 2020). Being more specific, increased ROS, on the one hand, causes modulation of NF-kB pathway leading to neuroinflammation via phosphorylation of NF-kB, modulation of activator protein-1 (AP-1), specificity protein-1 (SP-1), cAMP-responsive element-binding protein (CREB), and signal transducers and activators of transcription (STATs) (Rolle et al., 2020). Published evidence has also shown that increased ROS and proinflammatory mediators cause damage to the microvascular architecture, dysfunctional endothelial cells, and BBB disruption and neurotoxic manifestations (Niranjan, 2013). In various other published studies, it was found that exposure to radiotherapy causes lipid peroxidation and increased expression of COX-1/2 and prostaglandins (PGs) in the various region of the brain and found to be positively correlated with the increased level of TNF-α, IL-6, IL-1β, iNOS, intercellular adhesion molecule-1 (ICAM-1), monocyte chemoattractant protein-1 (MCP-1), vascular cell adhesion molecule-1 (VCAM-1), E-selectin etc. leading to neuroinflammation and cognitive dysfunction (Nizamutdinova et al., 2009; Jenrow et al., 2010; Choi et al., 2016).

Reduced neurogenesis was found to be associated with increased expression of caspase-3, c-jun, ERK ½ and mitochondrial dysregulation (Ji et al., 2020). Studies have also shown that exposure to radiotherapy causes increased expression of NMDA-R in the hippocampus, resulting in excitotoxicity and cognitive dysfunction (Lei et al., 2006; Franco-Pérez et al., 2020). It should also be noted that apart from increased oxidative stress, neuroinflammation, reduced angiogenesis. Reduced angiogenesis is also a decisive factor for radiotherapy-induced neurotoxic manifestations. Among various proangiogenic factors, vascular endothelial growth factor (VEGF) plays a pivotal role in this process (Imaizumi et al., 2010). Mechanistically, VEGF is accountable for the proliferation and migration of endothelial cells and the production of newer blood vessels. Moreover, reduced endothelial cells were associated with the reduced density of blood vessels and VEGF also play an important role in protecting endothelial cells from apoptosis and, hence, regulating the optimum angiogenesis (Loncaster et al., 2000).

Thus, considering the broad view, concluded that, as of now, the reported work was only focused on the details of brain-damaged-induced cognitive dysfunction and available repurposed pharmacotherapeutic agents. We found a lacuna of mechanistic representation of neurotoxic manifestation in radiotherapy-induced brain injury, shortcoming of available therapeutic regimen, use of alternative therapeutic approaches such natural products and their nano carrier-based drug targeting and the emerging role of miRNA based therapy to manage and treat radiotherapy-induced neurotoxic manifestations.

Thus, this manuscript discusses the mechanistic representation of brain injury, available Pharmacotherapy, their shortcomings, the neuroprotective role of natural products, their nanocarrier-based drug delivery, the proposed mechanism of neuroprotection and the emerging therapeutic promise of nanocarrier-based drug delivery to ameliorate radiotherapy-induced neurotoxic manifestations.

## 3. Brain injury and exposure to radiation

As discussed previously, radiotherapy is an important therapeutic regiment in managing and treating brain tumors (Flores-Castro & Sebastian-Barajas, 2021). Radiotherapy is often considered as first-line therapy in the treatment. Brain injury owning to radiotherapy is now considered as unavoidable circumstances and used after careful evaluation of risk-benefit ratio (Flores-Castro & Sebastian-Barajas, 2021). Ideally, a high dose of radiotherapy, i.e. 50–60 Gy in a divided dose, is used for the whole-brain or at a specific area, depending upon the pathogenesis (Warrington et al., 2012). Acute brain injury is reversible and initially characterized by edema and emesis (Sourati et al., 2017). However, late brain injury is considered an irreversible event associated with endocrinopathy and cognitive dysfunction and is a major concern for health care professionals (Sourati et al., 2017). Consequences of endocrinopathy are hypothyroidism, alteration in the gut-brain axis, growth retardation, cardiovascular consequences because of alteration in glucocorticoids and mineralocorticoids, gonadal damage etc (Sultana et al., 2020). Cognitive dysfunction, IQ impairment, and dementia are considered the late sequel of radiotherapy and more often affect patients’ quality of life. Ideally, patients suffering from brain tumors are considered as an ideal subject for the study of radiation’s impact on brain health and QOL (Turnquist et al., 2020). However, these types of studies are challenging because of the short follow-up period, high mortality rate, coexisting psychological status and a higher rate of social withdrawal. Still, studies conducted so far have profoundly emphasized the deleterious effect of radiotherapy on brain health and reduced QOL (Turnquist et al., 2020). Meyer’s et al. have reported reduced cognitive function among the patients when treated via paranasal irradiation with a total dose of 60 Gy in a divided dose of 1.8–2 Gy (Meyers & Brown, 2006). The study’s outcome showed that more than 80% of patients suffer from a learning disability, whereas 50% of patients reported difficulty in visual and motor functions (Meyers & Brown, 2006).

Similarly, severe cognitive dysfunction was found after 10 years when children were irradiated for the treatment of head and neck tumors (Chong et al., 2002). Sometimes patients with lung cancer are also exposed to radiotherapy to inhibit brain metastasis. In one of the long follow-up studies, when children with a confirmed report of acute lymphoid leukemia were exposed with 24 Gy radiotherapy and methotrexate, marked reduction in cognitive function, IQ score and learning, and memory deficit was observed found, confirming the late sequel of radiotherapy (Chan et al., 2001). In another study, with a follow-up of 15 years, it was found that exposure to 18 Gy was not associated with any neurobehavioral abnormalities (Greene-Schloesser and Robbins, 2012). Thus, it can be concluded that the extent of neurological dysfunction depends on the cumulate dose.

**Figure 2.**
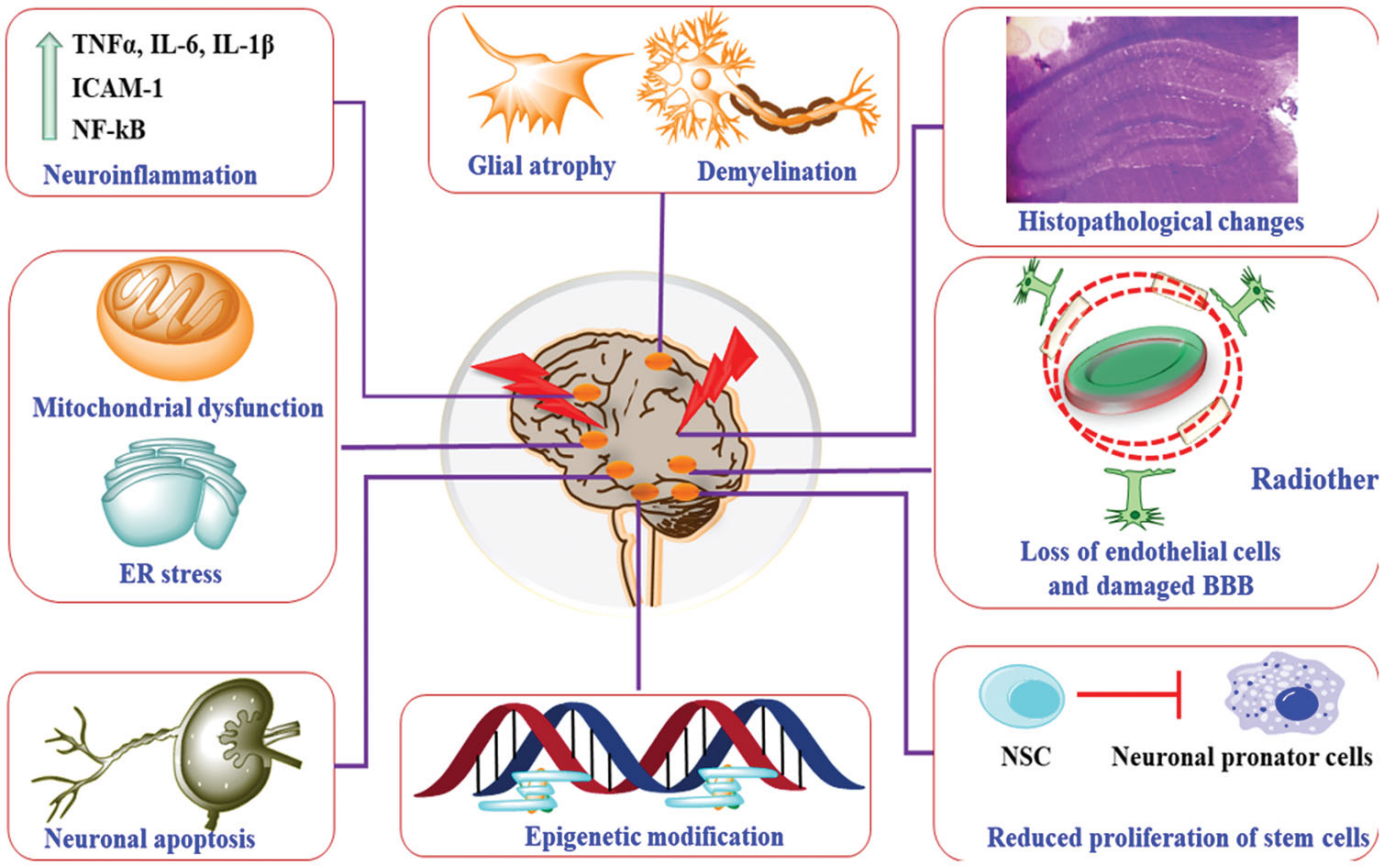
Pathophysiological presentation of radiotherapy-induced neurotoxic manifestations.

It is also important to understand that the studies mentioned above are based on the exposure of x-ray or gamma rays. Thus, recently, proton bean therapy (PBT) was explored as an alternative. PBT is now considered an emerging and novel radiotherapeutic technique with improved clinical efficacy and reduced toxicity (Turnquist et al., 2020; Raghavapudi et al., 2021). PBT imposes bean over a narrow area, and hence, adjacent tissues have remained unaffected. PBT is now extensively used in children because of more sensitive and irreversible changes in neurons (Hidaka et al., 2021). Head-to-head trials have also shown lesser neurobehavioral and neurotoxicological effects of PBT than X-ray and gamma-ray (Chambrelant et al., 2021; Plant-Fox et al., 2021) (Figure 2).

## 4. Clinical manifestation of radiation-induced brain injury

Clinical assessment and the manifestation of radiation-induced neurotoxicity is very important for timely treatment and management. Radiological examinations are commonly used to diagnose any adverse effect in the brain. CT scan, MRI, 3 D scan, Color doppler scan etc., are used (Chambrelant et al., 2021). However, these techniques have their own limitation and cannot be used on a routine basis. Thus, neurobehavioral or psychological tests are used to assess cognitive or behavioral dysfunction after radiotherapy. Ideally, mini-mental status examination (MMSE) is used to check irradiated patients’ cognitive function (Arevalo-Rodriguez et al., 2021). Apart from the MMSE, FACT-cognitive function test, profile mood assessment, patient health questionnaire, Hopkins verbal Learning Test, Rey-Osterreith Figure, Trail Making Test, Controlled Oral World Association Test, Digital Span Test, and California Verbal Learning Test are used for clinical assessment (Sultana et al., 2020). However, emerging evidence has shown insensitive reports of these tests and poor patient compliance and also this test is also not suitable for patients with a low education background. Thus, a battery-based assessment was developed that provides the assessment results within 30 min and is convenient for the patients (Ribeiro et al., 2020).

## 5. The pathological mechanism involved in the radiation-induced neuro dysfunctions

For a long time, inflammatory mechanisms and cytokines have played a pivotal role in neuronal damage and cognitive dysfunction. DAMP related to inflammasome activation, TLR-4 activation, and microglial activation is commonly reported after radiotherapy (Lumniczky et al., 2017). Additionally, radiotherapy has been reported to increase ROS and RNS that further initiate the inflammatory cascade via various integrated mechanisms.

In one of the published reports of the preclinical study, exposure of whole brain radiation (25 Gy) to the 6 months rat were analyzed for cognitive dysfunction, neuroinflammation, histopathological and immunohistochemical aberrations (Lee et al., 2012). The outcome of the study showed that radiotherapy-induced significant reduction in the behavioral function increased the markers of neuroinflammation, induced gliosis, and caused histopathological as well as immunohistochemical aberrations leading to Alzheimer’s disease-related pathology (Lee et al., 2012). In other published literature, exposure to 45 Gy radiation to rats causes a marked decline in behavioral functions, spatial learning, and refractory memory when assessed by the Morris water maze (MWM) (Lei et al., 2006). The study’s outcome also showed that radiotherapy caused increased expression level of NMDA receptors in the CA1 region of the hippocampus. However, no change in AMPA and synaptophysin expression level was observed (Lei et al., 2006). Limoli et al. (2004) showed that exposure to radiotherapy showed increased neuronal apoptosis increased the level of ROS and phosphorylation of Trp53, leading to inhibition of neurogenesis and cognitive dysfunction (Nieblas-Bedolla et al., 2021). Similar to Limoli et al. (2004), Collins-Underwood et al. (2008) showed the involvement of persistent oxidative stress via modulation of NADPH oxidase (Collins-Underwood et al., 2008). Additionally, radiotherapy caused increased expression of NF-kB, ICAM-1, and PA-1, leading to neuronal stress and neuroinflammation (Collins-Underwood et al., 2008).

Radiotherapy-induced reduced angiogenesis is another critical pathological attribute that leads to reduced cognitive function and severely affects patients’ quality of life (Lupattelli et al., 2020). In an exciting experiment, 10 Gy radiation was given to the rats and analyzed for the markers of angiogenesis. The study’s outcome showed reduced expression of VEGF, Ang-1, and Tie-2, leading to reduced endothelial cell proliferation and increased apoptosis (Lupattelli et al., 2020). Similar findings were also reported by Wei et al., 12 and Fukuda et al. (2005). It is well known that BBB performs the critical function of protecting the whole brain from various noxious stimuli. Disruption of BBB is a well-established pathological event. Surprisingly, Liu et al. (2010) showed that exposure of 10, 20 and 40 Gy radiation showed dysfunctional cognitive attributes, histopathological aberrations, and damage to the BBB (Li et al., 2021). Furthermore, Rola et al. (2004) showed that exposure of 2–10 Gy radiotherapy caused a significant reduction in the neurogenesis, activation of microglia, infiltration of monocytes, chronic inflammation, and hippocampal related memory dysfunction (Flores-Castro & Sebastian-Barajas, 2021). Complex signaling molecules involved in radiotherapy-induced neuroinflammation and cognitive dysfunction are shown in Table 2 (Zhang et al., 2018).

**Table 2.** The role of various signaling molecules in ration induced brain damage.

Dose of radiotherapy	Mechanism involved	Signaling molecule involved	References
5–35 Gy/single cycle	Neuroinflammation	iNOS, COX-2, MCP-1IL-1β, ICAM-1, IL-6, MIP-2 and TNF-α,	(Kyrkanides et al., 2002)
10 Gy/single cycle		MCP-1, IL-1β and TNF-α	(Lee et al., 2010)
10 Gy/single cycle		COX-2, IL-1β, c-Jun, IL-6 and TNF-α	(Deng et al., 2012)
10 Gy/single cycle	Reduced neurogenesis	NG2, NeuN, GFAP and Ki-67 and Ki-67	(Monje et al., 2003)
2–10 Gy/single cycle		NG2, NeuN, DCX, GFAP and Ki-67 and CD68	(Rola et al., 2004)
10 Gy/single cycle	Increased oxidative stress	MDA	(Limoli et al., 2004)
1–10 Gy/single cycle		NOX-4, NF-kB, ROS and PAI-1	(Collins-Underwood et al., 2008)
10 Gy/single cycle 40 Gy/8 cycle	Increased extracellular matrix	TIMPs, MMPs and collagen type IV	(Lee et al., 2012)
10 Gy/single cycle	Reduced angiogenesis	VEGF, Tie-2, Ang 1 and 2	(Lee et al., 2011)
10 Gy/single cycle	Neuronal apoptosis / death of progenitor cells	AIF, p53, Caspase-3 and nitro tyrosine	(Pazzaglia et al., 2020)
2–10 Gy/single cycle		Annexin V and PARP	(Fukuda et al., 2005)

### 5.1. Radiotherapy induced oxidative stress DNA damage

As discussed previously, radiotherapy causes severe neuronal damage, induced ROS production, RNS, damaged stem progenitor cells, and inhibited neurogenesis and angiogenesis. Additionally, radiotherapy alters the calcium signaling pathway, produces MDA, causes lipid peroxidation, and produces free radicals that lead to neuronal apoptosis, neuroinflammation, and cognitive dysfunction (Lumniczky et al., 2017). On the one hand, radiotherapy induces oxidative stress, whereas on the other hand reduces the activity of antioxidant enzymes such as SOD, CAT, GSH, etc (Robbins et al., 2002). Mitochondrial activity is primarily responsible for producing endogenous ROS, and radiotherapy has been reported to trigger this process and cause robust production of ROS, leading to oxidative stress (Iqubal et al., 2020). Excessively produced ROS acts on the biomolecules such as DA, proteins, lipids and causes DNA fragmentation, apoptosis, and necrosis (Iqubal et al., 2020). Moreover, CNS is more prone to oxidative stress lipid peroxidation, has less antioxidant activity, and cumulatively exhibits a higher number of free radicals such as O_2_^−^ (Iqubal et al., 2018; Khan et al., 2020). Additionally, neuronal cells such as oligodendrocytes are rich in iron content and hence, undergo Fenton reaction where H_2_O_2_ and iron react and produce OH^−^ (Connor and Menzies, 1995). It was also found that radiotherapy causes radiolysis of water molecules leading to the formation of O_2_^−^, OH^−^ and H_2_O_2_ (Allen et al., 2020; Liu et al., 2021). Studies have also shown that radiotherapy causes direct damage to mitochondria, interferes the ETC, salters the ATP production that together manifested in the increased level of calcium, reduces mitochondrial permeability, the release of cytochrome C, formation of apoptosome and caspases leading to neuronal apoptosis, inflammation, reduced neurogenesis and cognitive dysfunction (Limoli et al., 2004; Wang et al., 2017).

Apart from oxidative stress and alteration in mitochondrial integrity, radiotherapy also causes direct damage to DNA or produces free radicals. After radiotherapy, Single-strand breaks (SSBs), Double-strand breaks (SSBs), as well cross-linked DNA are produced (Jeggo & Löbrich, 2006). Among this fragmentation, DSBs are considered the most lethal for the neurons and produced due to the damaged phosphate backbone (Goodarzi & Jeggo, 2013). Additionally, SSBs produced via direct or indirect radiotherapy also get converted into DSBs and, if not repaired, causes point or spontaneous mutation leading to a variable degree of neurotoxic manifestations. Studies have shown the activation of p52, p65 NF-kB, p21, and caspases that, in addition to oxidative stress, cause neuroinflammation, neuronal apoptosis, and cognitive dysfunction (Ward, 1990; Wang et al., 2020).

In one of the published reports, when X-ray (6-Gy) was used, a significant increase in the level of TBARS, alteration in the level of total protein, and non-protein were found in the cerebellum region of the brain (Manda et al., 2007). The study also showed that radiation exposure caused significant histopathological aberrations in the brain and hence signifies the oxidative stress in the brain (Manda et al., 2007). These pathological events reversed toward normal when co-treated with antioxidant molecules (Manda et al., 2007). Moreover, the increased oxidative stress was found to be positively correlated with reduced cognitive dysfunction. Similarly, when a single dose of T-radiation (6-Gy, 1.66 Gy/min) was used, a significant increase in the marker of lipid peroxidation (MDA) and reduced in the enzymatic activity of GSH was found in the brain tissue and hence signified the oxidative stress by radiotherapy (Manda et al., 2007).

When 6 Gy whole-body electron beam radiation was used on the mouse, a significant increase in the marker of oxidative stress and increased anxiety was found (Radhakrishna et al., 2017). In another study, when 6 Gy, X-ray (cranial radiation) was used on the mice, various biochemical parameters were analyzed after 30 days (Radhakrishna et al., 2017). The outcome of the study showed that radiotherapy causes a reduced level of microtubule-binding protein doublecortin (Dcx) and ki-67 which is a marker of neuronal proliferation (Radhakrishna et al., 2017). Additionally, radiotherapy causes an increase in the level of 4-HNE (4-hydroxynonenal). Hence, the present study showed increased oxidative stress and reduced neurogenesis, leading to cognitive dysfunction and poor quality of life (Manda et al., 2009).

In an *in vitro* and *in vivo* experiment, when 0, 1, 2, 5, and 10 Gy X-ray was used for 0–24 h and for 33 days, increased neuronal apoptosis and increased level of ROS were found in the brain (Limoli et al., 2004). Additionally, the study also showed the mechanistic role of increased phosphorylation of Trp53 and p21 that was positively associated with apoptosis, oxidative stress, and reduced neurogenesis that leads to cognitive dysfunction (Limoli et al., 2004). In another mechanism-based study (*in vitro*) to explore the neurotoxic effect of radiotherapy, a single dose of 1–10 Gy gamma radiation was used (Collins-Underwood et al., 2008). Exposure to radiotherapy in the study was found to be associated with activated NADPH oxidase, increased expression of NF-kB, ICAM-1, Nox4, and PA-1, and a significant increase in the level of ROS in the brain (Collins-Underwood et al., 2008).

Moreover, unlike previously published reports, in this study, radiotherapy-induced increase oxidative stress in the brain was effectively ameliorated by using the antioxidant drug. Hence, based on the numerous published reports, it was concluded that radiotherapy of different magnitude is associated with significant oxidative stress, behavioral dysfunction, and neuronal dysfunction (Jenrow et al., 2010).

### 5.2. Immune response to radiation, microglial activation and neuroinflammation

In normal conditions, inflammatory cascade and immunogenic reactions are considered a protective mechanism in eliminating pathogens and restoring the homeostatic brain function (Lumniczky et al., 2017). Neuroinflammation is one of the major hallmarks of radiation-induced neurological disorders (Ballesteros-Zebadúa et al., 2012). Various preclinical and clinical evidence has shown a positive correlation between neuroinflammation and cognitive dysfunction (Ali et al., 2020; Lei et al., 2020; Yang et al., 2020). Various factors trigger neuroinflammation. Exogenous factors such as infectious pathogenic invasion after disruption of BBB and endogenous factors such as cellular damage caused by radiotherapy are important contributors to neuroinflammation (Iqubal et al., 2021; Iqubal et al., 2022).

When the body is exposed to inflammatory cascade, significant damage to the tissues and cellular structure but being a potent regenerative component, these tissues regain their function and perform (Iqubal et al., 2021). Considering that brain neuronal cells have the poor regenerative ability and thus minimize the impact during inflammation, interactive mechanisms play a critical; role between CNS and immunogenic cells (Engelhardt & Ransohoff, 2005). The BBB-cerebrospinal fluid barrier, as well as tight junction, play a pivotal role in this mechanism. Mobilization and immune function of antigen-presenting cells (APCs) is restricted due to the reduced level of MHC-1 and low count of dendritic cells (DCs) (Engelhardt & Ransohoff, 2005). Microglial cells located in the parenchymatous tissues are prime spots for regulating the immune response in the CNS. Microglial cells share similarities with the DCs and macrophages and possess APCs-like properties (Md et al., 2021). Microglial cells have been reported to express MHC and DAMPs on their surface. Thus, they can sense the change in the microenvironment and presence of noxious chemicals, stimulus and eventually initiate the cascade of immunogenic reactions (Md et al., 2021).

It is also important to understand that due to the presence of anti-inflammatory cytokines and chemokines, these microglial cells remain inactivated and become activated in the presence of proinflammatory cytokines (Biber et al., 2007; Md et al., 2021). It is also important to understand that DCs are ideally present in the CNS but far away from BBB and BBB-CSF barrier and thus, in normal conditions, not involved in the immune reaction (Bulloch et al., 2008). However, upon microglial activation or due to the presence of death signals, these DCs gets activated and participate in the neuroinflammatory response (Bulloch et al., 2008). Persistent exposure to radiation has been well-correlated with neuroinflammation and cognitive dysfunction. Accumulating evidence has shown the role of microglial activation as a central player in the neuroinflammatory event (Kalm et al., 2009).

Microglial and astrocytic activation is considered the initial step in acute neuroinflammation (Chivero et al., 2021). Microglia and astrocytes are specialized to sense any noxious stimulus in the microenvironment (Chivero et al., 2021). When the brain is exposed to radiation, neurons are damaged, and soluble factors that are initially present on the neuron surface and inhibit the microglial activation are destroyed (Han et al., 2021). In normal conditions, microglial cells are specialized in removing cellular debris via their phagocytic activity. However, during irradiation, microglial cells and astrocytes regulate the level of MHC and proinflammatory cytokines such as ILs, TNF-α, CX3CL1, CCL3, macrophages, etc (Han et al., 2021). Activated microglial cells also increase the level of reactive oxygen species (ROS), reactive nitrogen species (RNS) and cause subsequent damage to BBB, leading to more rebuts inflammatory cascades (Biber et al., 2007). In response to irradiation, DCs get activated and translocate to the periphery of BBB and come in contact with CSF, interact with T cells, and activate the immune response and acute neuroinflammation (Kalm et al., 2009; Schmal et al., 2021). In one of the clinical studies, when children are exposed to radiotherapy, activated microglia produces proinflammatory cytokines and inhibits neurogenesis (previously discussed), disrupt hippocampal neuronal signaling pathways, and chronic neurobehavioral abnormalities. Various proinflammatory cytokines such as IL-6, IL-1β, TNF-α, CMP-1, CCl2/3, MIP-2, CXCL2 etc., have been reported after radiation exposure higher than 7 Gy (Lumniczky et al., 2017).

Moreover, activated microglial cells have been identified even after months after radiotherapy and hence confirm the chronic inflammation related to radiotherapy (Mizumatsu et al., 2003). Involvement of microglial activation after radiotherapy was confirmed when an inhibitor of microglia showed reduced neuroinflammation after radiotherapy (Mizumatsu et al., 2003). In one of the interesting studies, when rats (young and aged) were exposed to 10 Gy radiotherapy, a reduced number of immature neurons were found in young rats, whereas aged rats showed an increased percentage of activated microglial cells (Schindler et al., 2008). This, study indeed validated the fact that radiation exhibits differential neurotoxic patterns in young and aged brains, and young brains are more sensitive toward neurogenesis. In contrast, aged brains are more prone to microglial activation, neuroinflammation, and cognitive dysfunction (Schindler et al., 2008; Dietrich et al., 2008; Hua et al., 2012). Other findings have also shown that radiotherapy causes microglial activation via TLR4 and molecule high-mobility group protein 1 (HMGB1) pathways. When HMGB1 binds to the TLR4 receptors, which is located on the microglial surface, it becomes activated and causes neuroinflammation and cognitive dysfunction (Frank et al., 2016). In one of the exploratory in vitro studies, the use of 10 Gy γ-ray to the BV2 cells caused phosphorylation of c-jun, MAPK kinases ERK1/2 in microglial and showed microglial activated neuroinflammation (Deng et al., 2012). Similarly, exposure to WBI (10 Gy and 15 Gy) was found to be associated with a significant reduction in neurogenesis in the dentate gyrus via modulation of the angiotensin-II pathway (Jenrow et al., 2010).

When persistent radiation exposure is given, a cascade of chronic neuroinflammation begins (Camponogara et al., 2021). Chronic neuroinflammation occurs when the production of inflammatory cytokines surpasses their inhibition and when leucocytic infiltration exceeds its inhibition (Xiao et al., 2021). Apart from microglial activation related neuroinflammation, radiotherapy has been directly reported to induced neuroinflammation causes via increased production of proinflammatory cytokines, ROS and RNS and eventually causes neuronal damage, apoptosis, necrosis and histopathological damage leading to neurobehavioral and cognitive dysfunction (Pazzaglia et al., 2020). Various signaling pathways such as p38 MAPK, GSK-3β, c-jun, NF-kB, and molecules such as Nrf2, TNF-α, ILs, chemokines etc., play a pivotal role in the neuroinflammatory cascade (Iqubal et al., 2021; Iqubal et al., 2022). Radiotherapy has been reported to alter the endogenous immune response with the neuronal cells and parenchymatous cells, cause microglial activation, and increase macrophages and leucocytes’ infiltration (Constanzo et al., 2020). Moreover, radiotherapy has been reported to enhance the nuclear translocation of NF-kB that leads to persistent production of inflammatory cytokines, activate NLRP3 inflammasome, regulate the TLRs mediated neuroinflammation, and reduce the antioxidant activity Nrf2 (Mohamed & Said, 2021; Zhuang et al., 2021). When the brain is exposed to radiotherapy more than 1 Gy, DNA damage occurs and DAMPs are produced, which is sensed by the immune components via pattern recognition receptors (PRRs) such as TLRs (Zhuang et al., 2021). Studies have shown the release of HMGB1, heat shock proteins, and uric acids alter radiotherapy, and these components causes activation of NF-kB as well as TLR-4 and result in neuroinflammation (Amini et al., 2021). Studies have also shown that neuroinflammation is followed by the induction of apoptosis that inhibits neurogenesis inhibits the recruitment of neurons into the hippocampal circuit leading to cognitive dysfunction (Dewey et al., 1995; Belka et al., 2004; Amini et al., 2021). As discussed previously, exposure to radiotherapy causes acute or chronic neurotoxic manifestation (0–6 months) and are associated with vascular malfunctioning, gliosis, perivascular edema, neuronal necrosis, and neuroinflammation that reflects into other disease conditions such as seizure, encephalopathy, ataxia etc (Edelstein et al., 2017). Additionally, studies have shown that post-radiation complications cause increased production of proinflammatory cytokines that leads to persistent neuroinflammation and dementia (Tofilon & Fike, 2000). Published evidence have also shown that radiotherapy causes significant damage to the BBB, increases its permeability leads to the infiltrations of lymphocytes, gliosis, and demyelination (Kyrkanides et al., 1999). Based on the experimental findings, it was found that radiotherapy causes increased mRNA expression of NF-kB, ILs, TNF-α and other adhesion molecules (Chiang et al., 1997). As per the published report of Lee et al., 2010, and others, exposure to radiotherapy causes increased nuclear translocation of NF-kB, triggers the proinflammatory response of activating protein-1 (AP-1), and CERB within 8 h. of exposure (Lee et al., 2010). In other studies, exposure of 2–8 Gy radiation caused increased NF-kB expression within 30 min of exposure and validated the hypothesis of radiotherapy-induced acute neuroinflammation (Hwang et al., 2006). In one of the *In Vitro* studies, exposure of 10 Gy radiation causes an increased level of COX-2, TNF-α as well as IL-1β (Ramanan et al., 2008). Kyrkanides et al. (2002) showed that radiotherapy exposure significantly increased COX-2, TNF-α, iNOS, ILs, ICAM-1 and MMP-9 and caused neuroinflammation (Kyrkanides et al., 2002). According to Hong et al. (1995), exposure of 2–7 Gy WBI causes a significant increment in the expression level of TNF-α, iNOS, ILs, ICAM-1, glial fibrillary acidic protein (GFAP) within 4 h. of exposure and hence exhibited neuroinflammatory response (Hong et al., 1995). Similarly, exposure of 0, 5, 15, 25 or 35 Gy gamma radiation causes increased expression of ICAM-1 and CD5 from 6 h onwards and continued for 7 days. The increased expression of these proinflammatory markers were positively correlated to the leucocytic infiltration and brain injury in a dose dependent manner (Olschowka et al., 1997). Moreover, Kim et al. (2002) reported that exposure of 10 Gy dose of gamma rays to the rats causes increasd expression of TNF-α and TGF-β leading to chronic inflammation (Kim et al., 2002). In one of the interesting study, by Gaber et al. (2003), exposure of single and fractioned dose of 2, 10 or 20 Gy radiation was associated significant neuroinflammation (Gaber et al., 2003). It was seen that after 20 Gy radiation expression level of ICAM-1 and TNF-α was 14- and 11-fold, respectively (Gaber et al., 2003). In one of the recently published literatures by Constanzo et al. (2020), exposure of 0–150 radiation to the rats showed damaged integrity of endothelial cells, histopathological damage, leucocytic infiltration, brain edema and showed anxiety-depressive like behavior (Constanzo et al., 2020).

### 5.3. Radiotherapy induced BBB damage

As discussed previously, BBB plays a pivotal role in maintaining the homeostatic integrity of CNS and prevent the entry of toxins as well as inflammatory cytokines into the brain. Studies have shown disruption of BBB, damaged endothelial cells, and tight junction after radiotherapy (Gorbunov & Kiang, 2021). Preclinical studies have shown the involvement of the ASMase pathway in BBB disruption after radiotherapy (Li et al., 2003; Wong & Van der Kogel, 2004). In one of the studies, the use of 20–40 Gy (single cycle) was associated with acute brain injury, BBB disruption, histopathological damage, and cognitive dysfunction (Peng et al., 2021). In another study, the use of 20–40 Gy radiotherapy disrupted BBB and causes neuroinflammation (Turnquist et al., 2020).

In a preclinical, experimental study, the use of 10, 20, and 40 Gy gamma radiation (300 cGy/min) for 60 days was found to be associated with significant damage to the BBB integrity (Liu et al., 2010). The outcome of the study showed that the use of 20–40 Gy radiation causes increased water content of the brain, caused significant histopathological damage in the cortex region of the brain (Liu et al., 2010). The damage of BBB was further validated by using Evans-Blue dye its increased level signifies the extent of BBB damage (Liu et al., 2010). As it is well established that MMPs and TIMP-1 play a vital role in maintaining BBB permeability, their increased level is directly correlated with the damaged BBB integrity and permeability (Lee et al., 2012). Therefore, an exploratory *in vivo* study was performed where a single dose of 10 Gy and 40 Gy fractional dose of gamma radiation was used (Lee et al., 2012). After 24 it was found that the expression level of MMPs and TIMP-1 was significantly increased, whereas the level of collagen IV was reduced significantly reduced in brain tissue and hence confirmed the damaged BBB (Lee et al., 2012). When 4–25 Gy gamma radiation was used and accessed after 3 days and 8 days for damaged BBB permeability (Fauquette et al., 2012). The outcome of the study showed that treatment with both doses causes double-stranded DNA damage in the endothelial cells and causes BBB damage which was confirmed by the use of fluorescence technique (Fauquette et al., 2012). In another interesting study, when a single dose of 60 Gy gamma radiation was used on the rats and analyzed over 2, 6, 12, and 24 weeks, significant disruption of BBB, vasculature leakage, loss of network of blood capillary, and atrophy in the cortex and necrosis in the white matter along with histopathological and ultrastructure was found (Rubin et al., 1994). When gamma radiation (0, 5, 10, 15, 25 and 35 Gy) was exposed on the right side of the brain hemisphere and analyzed over 4, 24, and 48 h and on 7th days for damaged BBB (Olschowka et al., 1997). Outcome of the study showed that exposure to the gamma radiation was associated with leucocytic infiltration and increased expression of ICAM-1 in the brain and hence signifies the disruption of BBB upon radiation exposure in a dose dependent manner (Olschowka et al., 1997).

**Figure 3.**
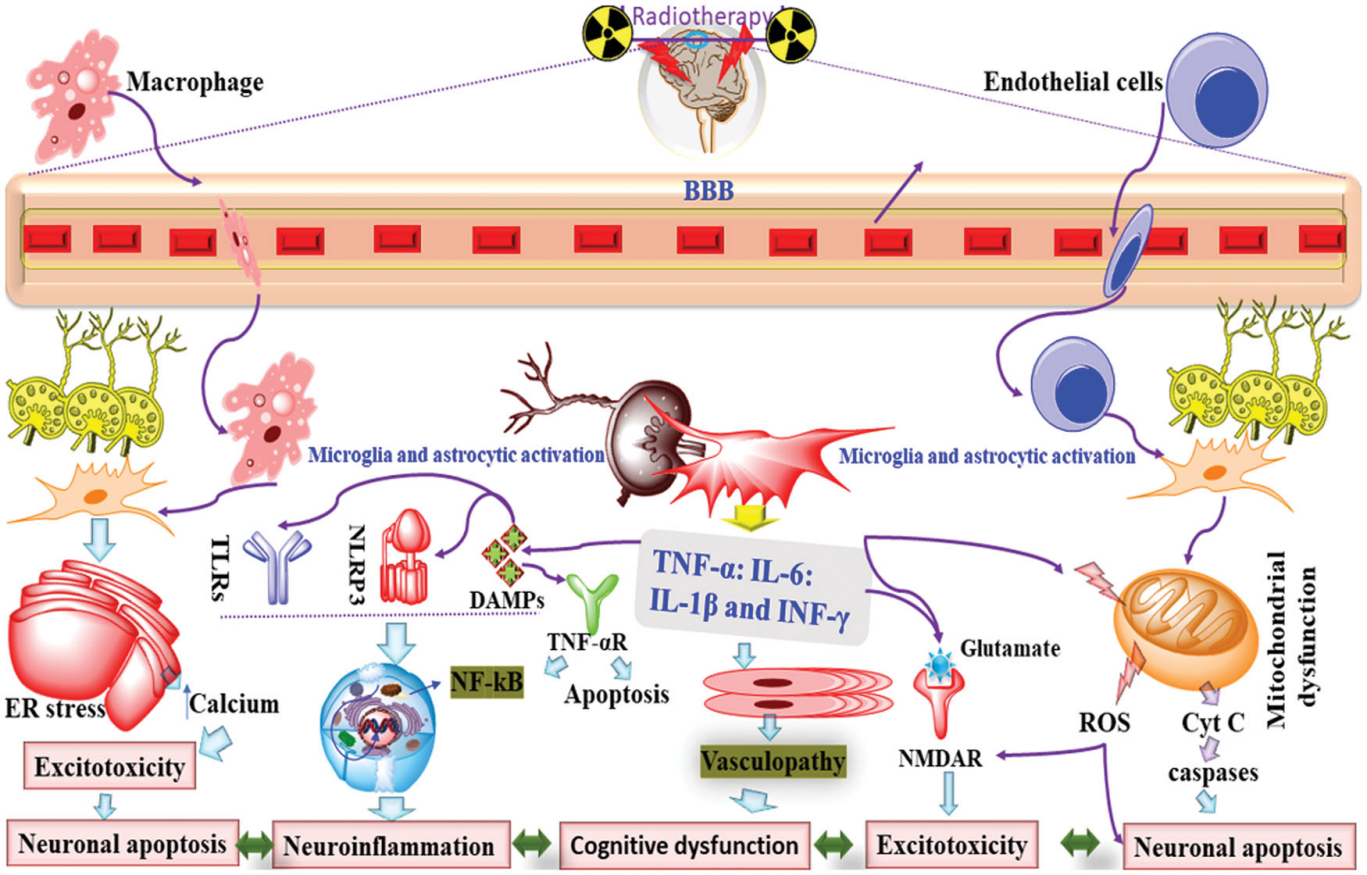
The neuroinflammatory aspect of radiotherapy.

The mechanism behind radiotherapy-induced BBB disruption and enhanced permeability was linked to the damaged endothelial cells after endothelial apoptosis (Li et al., 2003; Zhao et al., 2016). Furthermore, after WBRT >2–8 Gy, disruption of BBB increased DNA fragmentation, and reduced DNA repairing mechanism was found, leading to enhanced production of ROS, RNS, IL-6 and TNF-α (Rochfort & Cummins, 2015; Khan et al., 2017). Studies have also shown that radiotherapy also causes endothelial damage and neuroinflammation without affecting the integrity of BBB and causes radiotherapy-induced increased ICAM-1, VCAM-1, as well as P selectin in the brain (Kyrkanides et al., 1999; Sharp et al., 2003) (Figure 3).

### 5.4. Radiation and inhibition of neurogenesis and angiogenesis

Neurogenesis is one of the key regulatory mechanisms for maintaining normal neuronal functions such as synaptic plasticity, learning memory, and cognitive function. Neurons are ideally generated from the neuronal stem cells and progenitor cells that are mainly found in the hippocampus. Neurons produced in the DG region of the hippocampus get recruited into the neuronal circuit of the hippocampus and regulate learning and spatial learning (Wang et al., 2017). It has been reported that age-related cognitive dysfunction is positively related to the reduced rate of neurogenesis. Infect in the neurodegenerative disorders, reduced neurogenesis have been reported. Preclinical studies have shown the association between increased neuronal apoptosis, reduced neurogenesis, and radiotherapy (Guo et al., 2019; Ji et al., 2020; Gorbunov & Kiang, 2021). Indeed, studies have also shown that when lowering clinically used doses, i.e. 2–5 Gy are used, neurogenesis is inhibited within 48 h (Deng et al., 2012). When 10-Gy of whole brain radiation (gamma-ray) was used among the rats, the extent of neurogenesis was estimated using the bromodeoxyuridine immunofluorescence technique and expression of ki-67 and CD8 after 2 months of exposure (Ramanan et al., 2008). The outcome of the study showed that radiotherapy caused a significant reduction in the proliferation and angiogenesis in the subgranular zone and hence reduced the quality of life (Ramanan et al., 2008). When a single dose of 10-Gy X-ray was used among the rats, a significant increase in hippocampal inflammation and neuronal apoptosis was found and was found positively correlated to the reduced neurogenesis and angiogenesis (Monje et al., 2003). When mice were exposed to 2–10 Gy X-ray whole-brain irradiation and assessed for effect on neurogenesis, it was found that radiotherapy caused a reduced number of immature neurons and also reduced the formation of new neurons (Quimby and Luong, 2007). Additionally, exposure to X-rays caused microglial activation and reduced cognitive function (Quimby and Luong, 2007). In one of the exploratory studies, the use of 2–0 Gy gamma radiation (single dose) caused an increased level of proinflammatory cytokines and reduced neurogenesis as well s cognitive function after 1 day, 7 days, and after 12 weeks (Conner et al., 2011). These effects were significantly ameliorated by the use of RAAS and hence signified the role of this RAAS in radiotherapy-induced altered neurogenesis (Conner et al., 2011). Similarly, the use of 10–15 Gy whole-brain irradiation caused a marked reduction in the neurogenesis in the DG region of the hippocampus and was confirmed with the immunohistochemistry of ki-67 and CD68 cells (Jenrow et al., 2010).

The mechanism-based study revealed the role of JNK activation after radiotherapy that eventually stimulated apoptosis and inhibited neurogenesis (Deng et al., 2012). Additionally, these events were also associated with neuroinflammation and ROS and RNS. Hence, exercise and the use of anti-inflammatory and antioxidants drugs were found to be effective against reduced neurogenesis.

Interestingly, it was found that a high dose of radiotherapy causes demyelination and histopathological damage, whereas a low dose is more related to inhibition of neurogenesis and cognitive dysfunction (Deng et al., 2017). Although the exact mechanism of cognitive dysfunction in radiotherapy is not clear, it was hypothesized that inhibition of neurogenesis, alteration in long-term potentiation, and discrete release of neurotransmitters are major confounding factors (Pazzaglia et al., 2020). Apart from neurogenesis, angiogenesis is also a critical factor for the maintenance of normal brain health. Angiogenesis refers to the formation of new blood vessels from the preexisting blood vessels (Li et al., 2021). Appropriate capillary density is crucial for the required supply of blood, oxygen nutrients, neurochemicals, trophic factors, and neurohormones (Lee et al., 2012). Any change in angiogenesis or reduction in capillary density is directly related to neuronal dysfunction and other neurotoxic consequences. Reduced capillary density, microvasculature or reduced angiogenesis is a hall mark of various neurodegenerative disorders such as AD or PD and correlated to cognitive dysfunction (Storkebaum et al., 2004). Radiotherapy (5–200 Gy) has been reported to damage the microvasculature, induce apoptosis, and reduce angiogenesis (Pazzaglia et al., 2020). Additionally, radiotherapy has been reported to cause dysfunction endothelial proliferation that results in the formation of blood vessels with abnormal shape size and diameter, which eventually leads to the reduced blood supply and causes hypoxia and cognitive dysfunction (Zhang et al., 2015). In one of the studies, the use of 40 Gy metronomic doses for ten weeks resulted in a reduced diameter of blood vessels, cognitive dysfunction, and dementia (Brown et al., 2005, 2007).

## 6. Neuroprotective potential of natural products against radiotherapy

**Figure 4.**
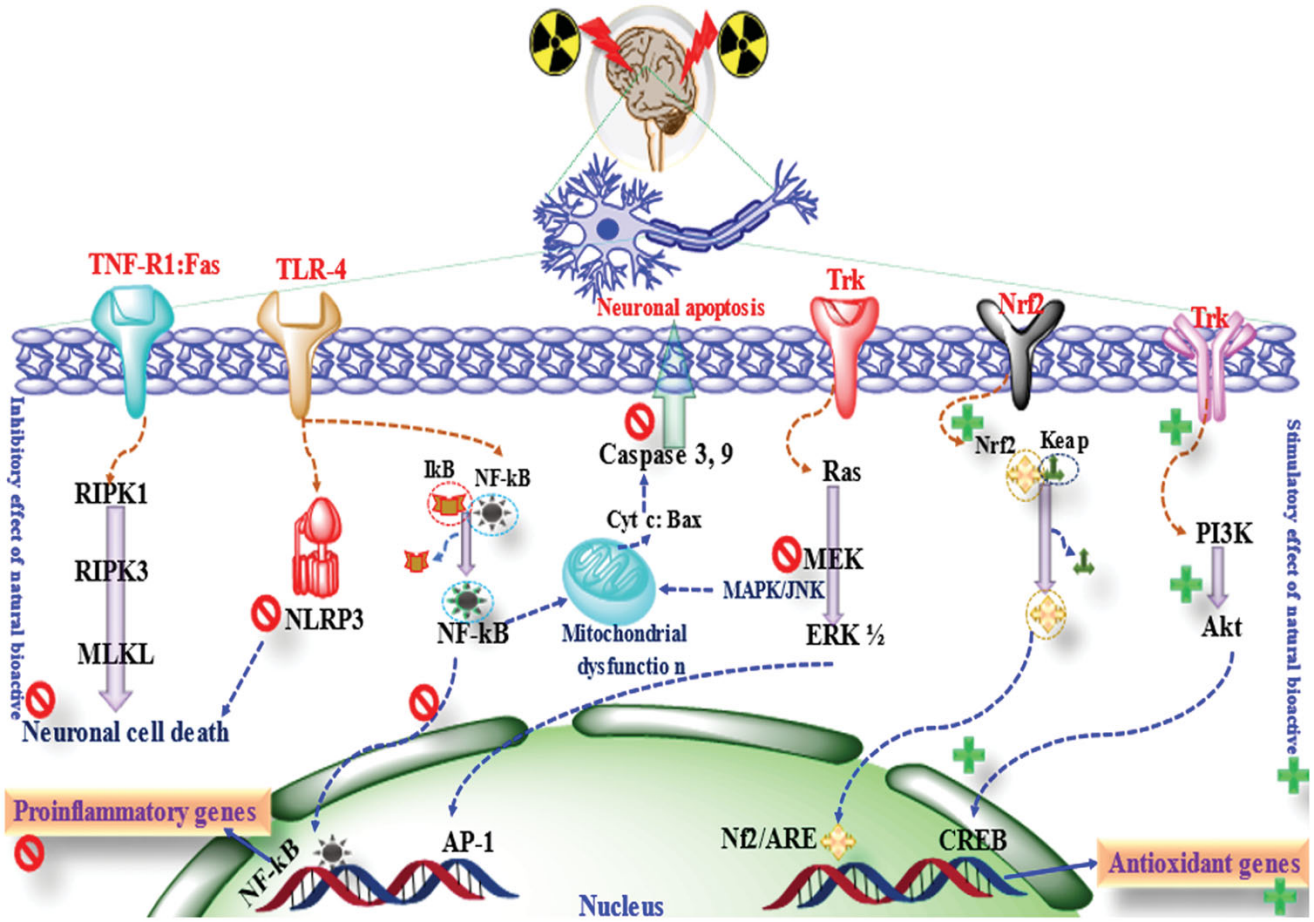
The mechanism of neuroprotection by phytoconstituents.

For a long time, natural products have been extensively used in various disease conditions. Considering neurological disorders, natural products have shown great potential in management and treatment. Since radiotherapy induces significant oxidative stress, neuroinflammation, neuronal apoptosis, mitochondrial dysfunction, inhibits neurogenesis and angiogenesis, the available natural products have been reported to counteract this neurotoxic manifestation. The natural product follows ‘one drug multiple target hypotheses. Natural products exhibit antioxidant activity via scavenging of produced ROS, inhibit ROS production, xanthine oxidase, cyclooxygenase, lipoxygenase, etc (Iqubal et al., 2020; Iqubal et al., 2021; Tuli et al., 2021). Flavonoids have been reported to directly interact with the OH- group of various semis quinone substances and eventually terminate the chain reaction (Wang et al., 2020). Natural products also activate the Nrf2 pathways and increase the endogenous antioxidant defense system, leading to an increased antioxidant effect (Iqubal et al., 2021). Natural products also inhibit the neuroinflammatory cascades, prevent DNA damage, and stimulate DNA repair mechanisms (Iqubal et al., 2021) (Figure 4).

Various phytoconstituents such as naringenin, EGCC, genistein, quercetin troxerutin have been documented to prevent DNA damage against radiotherapy (Peng et al., 2018). Various phytoconstituents have been explored for possible neuroprotection after radiotherapy. When quercetin was used at the dose of 50 mg/kg for 15 days against 20 Gy radiotherapy, a significant reduction in the level of MDA, inflammatory mediators, neuronal degradation was observed and thus signifies the neuroprotection (Kale et al., 2018). When quercetin was studied in the in vitro set up with 2 Gy gamma radiation, reduction in ER stress, TNF-α, markers C/EBP-homologous protein (CHOP), JNK and increased in the level of Tuj1 protein was found, and thus signifies the neuronal survival and neuroprotective potency of this drug (Kale et al., 2018; Chatterjee et al., 2019). When the same molecule was studied in combination with rutin for 3 weeks against 5 Gy radiation, reversal of Nrf2, PI3K, AKT, and free radicals toward the baseline was found (Thabet & Moustafa, 2018). When 5, 7-dihydroxyflavone (DHF) was administered at the dose of 50 mg/kg for 3 weeks against 5 Gy radiotherapy, reduction in MDA level, acetylcholinesterase and cysteine aspartic proteinase-3 were found (Mansour et al., 2017). Baicalein is a well-known natural product and exhibits a polyvalent mechanism of action. When this drug was used at the dose of 10 mg/kg for seven days against radiotherapy, a significant reduction in neuronal cell death, neuronal progenitor cells, stimulation of neurogenesis, and improvement in learning and memory was found (Oh et al., 2013). EGCC, when used at the dose of 2.5 and 5 mg/kg, for three continuative days, against 3 Gy radiotherapy, reduced level of TNF-α, Aβ, IL-6 and increased level of antioxidant enzymes such as SOD, CAT and GSH were found (Frank et al., 2016). Additionally, EGCC treatment inhibited the DNA damage, reduced the expression of p53, Bax, caspases and increased the expression of Bcl-2 and hence exhibited antiapoptotic and neuroprotective effects (El-Missiry et al., 2018). Wogonin is yet another well-studied phytochemical with multiple health benefits. When wogonin was used at the dose of 30 mg/kg for 15 against radiotherapy, reduction in the level of proinflammatory cytokines such as TNF-α, IL-1β, IL-6 and elevation in the level of SOD, CAT, GSH, GPx, Nrf2 were found. Additionally, significant reduction in the neurodegeneration was found upon wogonin administration (El-Missiry et al., 2018). Astragalus have been reported to reduce the NO level in the brain and improved the cognitive dysfunction when exposed to radiotherapy (Xiao & Chen, 2005). Astragalus also reversed the neuronal damage, inhibited apoptosis, stimulated neurogenesis and improved learning and memory (Xiao & Chen, 2005). In another experiment, exposure to 30 Gy causes visual loss and cognitive impairment where as treatment with astragalus improved the visual disability and cognitive dysfunction (Tang et al., 2010; Ji et al., 2015). Salvia Miltiorrhiza whole extract and its bioactive constituent tanshinones and depsides have been reported to improve the structural and functional outcome of CNS and improve patients’ quality of life (Yi et al., 2004). It was found that radiotherapy causes reduced ATPase and increased Na^+^, Ca^2+^ levels in the brain, resulting in neuronal damage and behavioral dysfunction. Treatment with Salvia Miltiorrhiz reversed the level of these components toward normal and improved the learning and behavioral abnormalities (Zhang et al., 1999). In another study, treatment with Salvia Miltiorrhiza reduced oxidative stress and reduced MDA and ICAM-1 in the brain (Mou et al., 2004; Duan et al., 2011). Broomrape is a well-known Chinese traditional medicine and is reported to have neuroprotective potential against radiation-induced neurotoxicity (Jiang et al., 2001). Radix Hedysari is also an extensively explored phyto herb, and studies have shown reversal of SOD, MDA, and other neurochemicals toward normal when exposed to radiotherapy (Li et al., 2010). When safflower was used to mitigate radiotherapy-induced neurotoxicity, the outcome of the study showed a significant reductio in SOD and MDA and improved the stability of BBB and cognitive dysfunction (Gan et al., 2012). Similarly, ginkgo, ginseng, Kang-fu-ling and shenqi have also been reported to inhibit oxidative stress, upregulate the Nrf2, inhibit neuroinflammation, reduce the level of NF-kB and improve cognitive dysfunction (Ding et al., 2013; Hu et al., 2014; Sun et al., 2014; Zhang et al., 2015) (Tables 3 and 4).

**Table 3.** The neuroprotective role of various natural bioactive against radiotherapy-induced neurotoxic manifestations (132).

Phytoconstituents	Dose of radiotherapy	Dose of phytoconstituents	Mechanism of action
Quercetin	20 Gy	50 mg/kg for 15 days	Reduced neuroinflammation and exhibited antioxidant effect
	2 Gy	5–100 µM	Reduced ER stress, C/EBP-homologous protein (CHOP), and TNF-α level
Baicalein	16 Gy	10 mg/kg for 7 days	Inhibited neuronal apoptosis, death of stem progenitor cells, and stimulated neurogenesis
EGCG	3 Gy	2.5 and 5 mg/kg for 3 days	Reduced the level of TNF-α, IL-6, IL-1β, DNA damage, and inhibited apoptosis.
Cyanidin	6 Gy	50, 100 and 200 mg/kg for 14 days	Improved in body weight, complete bound, and inhibition of leucocytic proliferation
Silymarin	0.2-0.6 Gy	140 mg/kg/	Mitigated the DNA damage
Genistein	8.75 Gy	200 mg/kg	Reduced DNA damage and inhibition of leucocytic infiltration
Rutin	5 Gy	200 mg/kg for 21 days	Reduced the activity of GSK-3β, PI3K, Akt, and increased Nrf2 activity
5, 7-dihydroxyflavone	5 Gy	50 mg/kg for 21 days	Reduced the MDA, Aβ, and caspases

**Table 4.** The neuroprotective role of various extracts against radiotherapy-induced neurotoxic manifestations (132).

Extract	Active constituent	Dose of radiotherapy	Mechanism of action
Astragalus membranaceus	Astragalus and saponin.	4.5 Gy and 30 Gy	Reduction in lipid peroxidation, MDA level, and improvement in learning and memory
Salvia Miltiorrhiza	Tanshinone and cryptotanshinone	22 Gy	Reduction in oxidative stress, ICAM-1 and improvement in learning and memory
Broomrape	Ergosterin and cistanche	5 Gy	Reduction in oxidative stress, improvement in immune component, and increment in neuronal viability
Radix Hedysari	Hedysarum	2 Gy	Reduction in oxidative stress
safflower	Carthamin and safflower yellow	4 Gy	Reduction in oxidative stress
Arnebiae Radix	Shikonin and acetyl shikonin,	0.5 Gy	Reduction in oxidative stress and improvement in learning memory
Ginkgo	ginkgolides	20 Gy	Inhibition of apoptosis, stimulation of neurogenesis, reduction of ROS and neuronal cell death
Ginseng	Ginsenoside	30 Gy	Inhibition of apoptosis, stimulation of neurogenesis, and protection of hippocampal neuron
Shenqi	Codonopsis and astragalus polysaccharides	20 Gy	Reduced BBB permeability, reduced level of TNF-α, IL-1β, reduced expression of p53, and improved learning and memory
Renal invigoration (978-1)	Icariin and lignin	20 Gy	Inhibit apoptosis and improve learning memory

## 7. Limitation of phytoconstituents and role of nanocarriers against the radiotherapy-induced neurotoxic manifestation

No doubt, phytoconstituents are potent therapeutic molecules against radiotherapy-induced neurotoxic manifestation. Phytoconstituents have been considered safe, economical, easily available, and proven health benefits for a long time. However, preclinical studies only explore phytoconstituents, and very few products have reached clinical trials. These phytoconstituents suffer from the limitation of stability during storage and after consumption. Being a potent antioxidant molecule, most phytoconstituents are prone to oxidative stress and sensitive to metal ions and environmental factors (Hong et al., 2002; Caritá et al., 2020). Thus, there is a major challenge to maintain the stability of these phytoconstituents to reach the target site without getting damaged or unstable. Another major challenge for phytoconstituents is their pharmacokinetic limitation. These products have low bioavailability, low solubility and undergo fast hepatic metabolism (Byeon et al., 2019; Nehal et al., 2021). Since intra cerebrovascular or intralesional administration or intravenous administration is difficult. Hence, the oral route appears to be an available option. Still, these drugs already have poor bioavailability, and the use of the oral route will eventually cause reduced systemic absorption and desired therapeutic outcomes (Byeon et al., 2019). Apart from the physiological limitation of these phytoconstituents, pharmacodynamic limitations also exist. The presence of tight junction in BBB, P-glycoprotein, efflux, breast cancer and multidrug resistance-associated protein located on the BBB surface restricted the entry into CNS and pumped out them in the adjacent lumen of blood vessels (Bruinsmann et al., 2019; Bicker et al., 2020). Studies have also explored the intranasal route for drug delivery into the brain, but increased clearance muscularly, mucosal irritation, and low intake volume are major obstacles (Hong et al., 2019).

**Figure 5.**
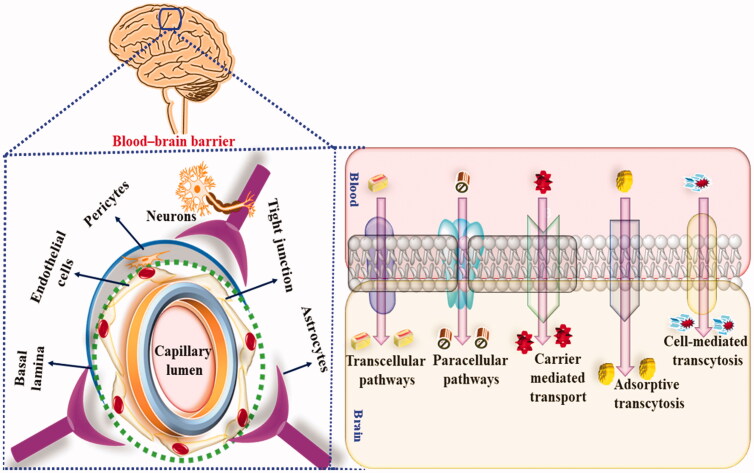
Showing the BBB permeation of nanocarriers in radiotherapy-induced neurotoxic manifestations.

Therefore, to overcome these pharmacokinetic and pharmacodynamic limitations, nano carrier-based drug delivery has been used. Nanocarrier-based drug delivery exhibit several advantages as compared to a conventional drug, such as increased stability, increased bioavailability, controlled release of the drug, bypass of hepatic metabolism, low dose, reduced side effects and maximum availability of the drug at the target site. Additionally, lipophilic nanocarriers easily cross BBB via precellular and transcellular transportation and exhibit the desired therapeutic outcome, as shown in Figure 5. Currently, polymeric NP, polymeric micelles, dendrimers, solid lipid nanoparticles (SLNs), liposomes, niosomes etc., have been used for the delivery of phytoconstituents into CNS to mitigate radiation-induced neurotoxic manifestations.

### 7.1. Polymer-based NPs

These are amphiphilic and self-assembled nanocarriers made up of hydrophobic and hydrophilic components where hydrophobic parts are located inside and hydrophobic components are located outside (Manek & Petroianu, 2021). The size of these nanocarriers ranges from 10 to 100 nm and can easily incorporate hydrophobic phytoconstituents to enhance the bioavailability and solubility (Manek & Petroianu, 2021). The presence of hydrophilic outer components such as PFG helps prolong circulation time in blood and allows the permeation across BBB (Avramović et al., 2020). In one of the recent studies, when CQ10 was encapsulated in the PEG polymeric nanocarriers, the bioavailability and pharmacological effect were significantly improved (Sikorska et al., 2014).

Currently, synthetic, natural, and neutral polymers are being to transport phytoconstituents across BBB. Poly (lactic-co-glycolic acid) (PLGA), polylactic acid (PLA), polyglycolic acid (PGA) etc., are some of the commonly used synthetic polymers to assist the transportation of various phytoconstituents across BBB (Calzoni et al., 2019). Tanshinone IIA is one of the phytoconstituents explored for the neuroprotective effect against radiation-induced neurotoxic manifestations. However, this bioactive suffers from pharmacokinetic limitations, and hence tanshinone IIA conjugated serum albumin polymeric nanocarrier was fabricated and explored for the possible neuroprotective effect (Calzoni et al., 2019). The study’s outcome showed significant improvement in bioavailability across BBB and exhibited potent anti-inflammatory and antiapoptotic effects (Liu et al., 2013).

Additionally, the presence of serum albumin in the formulation assisted in the transcytosis mechanism via interaction with the negatively charged cell membrane of endothelial cells (Liu et al., 2013). In another recently published ginsenoside, Rg3 and thioflavin T was conjugated with angiopep-2-PLGA and studied for the possible neuroprotective effect (Aalinkeel et al., 2018; Shi et al., 2020). The outcome of the study showed marked improvement in the bioavailability across the BBB. This nanocarrier approach showed an improved antiinflammatory, antioxidant and antiapoptotic effect in the brain. Additionally, the use of angiopep-2-PLGA was reported to interact with the low-density lipoprotein receptor-related protein, which is present on endothelial capillary cells and facilitates the uptake of phytoconstituents across BBB via endocytosis (Aalinkeel et al., 2018; Shi et al., 2020).

Intranasal drug delivery is another novel approach used for drug delivery into CNS. The intranasal route offers several advantages, such as bypassing hepatic metabolism and systemic adverse effect (Shi et al., 2020). By using this route, drugs directly reach cerebrospinal fluid (CSF) via the olfactory bulb. However, this route is again limited by several pharmacokinetic challenges, and hence, nanocarrier-based drug delivery of phytoconstituents via the intranasal route has been used to maximize neuroprotective potential. Ideally, mucoadhesive components are added to increase bioavailability (Shi et al., 2020). Apart from synthetic polymers, several naturally derived polymers are being used to enhance the CNS’s bioavailability and counteract neuroinflammation, neurodegeneration, and cognitive dysfunction. Polysaccharides such as chitosan, chondroitin sulfate etc. are currently used for brain targeting (Shi et al., 2020). These polymers are safe, biocompatible, biodegradable, offer a controlled release pattern, and enhance the circulation time leading to significant improvement in neuroprotection (Shi et al., 2020). Among various natural polysaccharides, CH is extensively used because of the presence of positively charged amine groups. Being positively charged, CH easily gets attached to the nasal cavity’s epithelial cells and facilitates the increased retention time in the nasal cavity, leading to improved BBB bioavailability (Nagpal et al., 2010). CH has also been reported to interact with the sialic acid present in the mucin and helps in the permeation across the olfactory bulb (Nagpal et al., 2010). Moreover, CH also possesses intrinsic antioxidant properties, scavenging free radicals and exhibiting a neuroprotective effect (Ngo and Kim, 2014). When piperine was used in conjugation with CH for the intranasal drug delivery, the study’s outcome showed enhanced antiinflammatory, antioxidant and antiapoptotic effects (Elnaggar et al., 2015). Similarly, when huperzine was used for the brain targeting in conjugation with CH, it was found that CH interacts with the lactoferrin receptors and assists in the endocytosis leading to increased permeation across BBB (Meng et al., 2018). Dendrimers are yet another polymeric nanocarrier and gained widespread attention for the targeted drug delivery across BBB and to counteract neuroinflammation and other neurotoxic manifestations. Poly (amidoamine) (PAMAM) is one of the commonly used dendrimers that easily undergo transcytosis and protects the encapsulated bioactive from lysosomal degradation via promoting endo-lysosomal escape (Srinageshwar et al., 2019).

### 7.2. Lipid-based nanocarriers

Lipid-Based nanocarriers such as solid lipid nanoparticles, nanostructured lipid carriers, and liposomes are also nan-range nanocarriers (100–400 nm) fabricated using solid lipids, liquid lipids, surfactants and co-surfactants (Iqubal et al., 2020). Recently, SLN and NLCs have been extensively used for the targeted delivery of phytoconstituents into the CNS because of their increased loading capacity, controlled release pattern, improved biocompatibility, and assured safety profile (Subramaniam et al., 2020). SLNs and NLCs have been conjugated with hydrophilic polymers such as PFG or polysorbates (Yasir & Sara, 2014). Various complexes have been prepared using ApoE that offers increased circulation time and assists in BBB permeation. Moreover, NLCs and SLNs are preferred for the intranasal drug delivery of phytoconstituents for neuroprotective potential (Bhatt et al., 2016). When astaxanthin SLNs were fabricated, the study’s outcome showed enhanced loading capacity, controlled release of the drug, increased bioavailability, the antioxidant and anti-inflammatory effects (Bhatt et al., 2016). Similarly, when curcumin-loaded NLCs were tested for neuroprotective potency, cellular uptake from BBB via endocytosis and improved neuroprotection were found (Meng et al., 2015).

Apart from SLNs and NLCs, liposomes are other lipid-based nanocarriers spherical in shape and mode up of phospholipid bilayers (Lai et al., 2021). Liposome offers the advantage of encapsulation of hydrophilic as well as lipophilic natural bioactive (Lai et al., 2021). Despite being an attractive nanocarrier, liposomes are removed from the circulation via endogenous reticuloendothelial system (RES) (Lai et al., 2021). Thus, several attempts such as reducing particle size, surface charged neutralization, and using polymers such as PEG were made to overcome the limitations (Wang et al., 2019). Andrographolide is a well-known natural bioactive with potent neuroprotective properties. However, it suffers from pharmacokinetic limitations of low bioavailability and hence, PEGylated cationic liposome as fabricated and studied for neuroprotective potential (Piazzini et al., 2018). When curcumin liposomal formulation was administered, significant improvement in bioavailability and reduction in neuroinflammation in CNS was found because of transferrin-mediated endocytosis (Papadia et al., 2017).

### 7.3. Inorganic NPs

Apart from polymeric and lipid-based nanocarriers, inorganic nanoparticles have been extensively used for the targeted drug delivery in CNS. Se, gold, iron oxide, carbon, albumin, and exosome are some of the commonly studied NPs to counteract neuroinflammation. Se is an endogenous antioxidant and has anti-inflammatory properties. Se possesses the intrinsic potential of enhanced drug loading capacity and is reported to be safe for brain targeting (Maiyo & Singh, 2017). In one of the exploratory studies, PEG-conjugated Se was found to be highly concentrated in the brain tissue and found to exhibit a potent neuroprotective effect (Yin et al., 2015; Amani et al., 2019). In another study, administration of B6 in conjugation with sialic acid and Se showed marked permeation across BBB and exhibited improved antioxidant, anti-inflammatory, and antiapoptotic effects and improved cognitive dysfunction (Yin et al., 2015). Gold nanoparticle is also explored for the possible neuroprotective potential because of ease of preparation, uniform size distribution, permeability across BBB, and assured safety profile (Sonkar et al., 2021). When anthocyanin in conjugation with Peg and gold NPs was explored for the possible neuropeptide effect, it was found that these NPs exhibited improved bioavailability, antiinflammatory, antioxidant and anti-apoptotic activity in the brain tissue (Kim et al., 2017; Li et al., 2019). Iron Oxide NPs are also extensively studied nanocarriers because of ease of preparation and uniform size distribution. Osmotin is a protein isolated from tobacco and, when fabricated using iron-oxide NPs, showed marked antioxidants and neuroprotective effects (Amin et al., 2017; Sonkar et al., 2021). Similarly, carbon nanotubes, albumin NPs, and exosomes have been studied for promising nanocarriers to deliver natural bioactive to mitigate neuroinflammation and cognitive dysfunction (Amin et al., 2017).

### 7.4. Recent advancements in the delivery of phytoconstituents across BBB to reduce neuroinflammation and cognitive dysfunction

Active brain-targeting is one of the approaches to enhance the transportation of phytoconstituents across the BBB. Active targeting involves the binding of drug components loaded into the nanocarrier ad their interactions with the various proteins or receptors using an external magnetic field or ultrasounds (Teleanu et al., 2018). This approach has targeted the specific inflammatory cells or neuronal structure to counteract neuroinflammation and associated cognitive dysfunction (Teleanu et al., 2018). Neutrophilic is one of the neuroinflammatory cascades’ inflammatory components and is responsible for oxidative stress and cognitive dysfunction. To inhibit the role of neutrophils in neuroinflammation, phytoconstituents conjugated with proline-glycine-proline peptide were used so that this complex binds to CXCR2, which is located on the surface of neutrophils (Li et al., 2018; Dang et al., 2019). Nanovalve is another advancement in brain drug delivery to treat and manage neuroinflammation. Cyclodextrin and ferrocene are some of the nano valvular systems which was used owning to the positively charged surface to reduce oxidative stress (Sun et al., 2019) (Table 5).

**Table 5.** Phytoconstituents-based nanocarrier to manage radiotherapy-induced neurotoxic manifestations (192).

Phytoconstituents	Nanocarrier	Conjugation	Mechanism of neuroprotection
Coenzyme Q10	Polymeric nanocarriers	PEG-and tocopherol	Coenzyme Q10 nanocarriers enhanced the bioavailability and pharmacotherapy
Tanshinone IIA		PEG-PLA	Improved uptake into the brain via endocytosis
Rg3 and thioflavin T		Angiopep-2 and PLGA	Nanocarriers permeate the drug across BBB and mitigate the glial cells mediated neuroinflammation
Urocortin		Odorranalectin, PEG and PLGA	Mucoadhesivness improved the bioavailability of the brain and exhibited neuroprotection.
Piperine		Tripolyphosphate	A positively charged surface potentiated the mucoadhesiveness and showed the neuroprotective effect.
Huperzine A		lactoferrin	Positively charged surface and endocytosis increased the bioavailability in CNS
N-acetyl cysteine		PAMAM dendrimers	Increased cellular uptake, antioxidant and anti-inflammatory effects were responsible for neuroprotection.
Astaxanthin	Lipid-based nanocarriers	SLNs	Enhanced loading capacity and drug accumulation in neurons exhibited neuroprotection.
Curcumin		Lactoferrin	Increased permeation across BBB synergized neuroprotection
Curcumin derivative		Lactoferrin and ApoE	Lactoferrin and ApoE increased permeation across BBB synergized neuroprotection
Se	Inorgenic nanocarriers	B6 peptide	Increased uptake across and intrinsic antioxidant, as well as anti-inflammatory effects, were responsible for neuroprotection.
Anthocyanin		PEG	Increased uptake across and intrinsic antioxidant, as well as anti-inflammatory effects, were responsible for neuroprotection.
Osmotin		Dextran	Magnetic targeting overcome the pharmacokinetic limitations and increased neuroprotective effect
Berberine	Carbon nanocarriers	polysorbate 80	polysorbate 80 improved the biocompatibility and bioavailability, and pharmacotherapy
Gallic acid	Biomimetic nanocarriers	Exosomes	Exosomes improved the solubility, bioavailability, neuronal uptake, and neuroprotection

## 8. Conclusion

The current review article discussed the cellular and molecular pathogenesis in the CNS after exposure to radiotherapy, which was intended to manage and treat brain tumors and other diseased conditions. Based on the preclinical and clinical findings, it has been reported that exposure to radiotherapy causes neuroinflammation and neurotoxic manifestations that eventually lead to alteration in hippocampal microenvironment as well reduced neurogenesis (Brown et al., 2013). In the present manuscript, we discuss the mechanistic representation and signaling pathways involved in radiotherapy-induced brain damage and cognitive dysfunction, but as of now, the exact mechanistic pathway is not clear. Studies have shown the involvement of neuronal oxidative stress, neuroinflammation, microglial activation, reduced neurogenesis, angiogenesis, and altered release of neurotransmitters, as a consequence of radiotherapy (Kim et al., 2008). Studies have also shown leads to altered synaptic plasticity and cognitive dysfunction (Wong & Van der Kogel, 2004). It also important to understand that apart from the direct neurotoxic manifestation of radiotherapy, use of chemotherapeutic drugs among cancer patients and immunological dysfunction because of carcinogenesis also significantly exhibit cognitive dysfunction (Iqubal et al., 2020). We have also reported the marked neuroinflammation, oxidative stress, apoptosis, histological damage, increased expression of NF-kB, NLRP3, and MPO upon administration of cyclophosphamide in Swiss albino rats (Iqubal et al., 2019; Iqubal et al., 2020). Thus, whenever radiotherapy is combined with pharmacotherapy, utmost precaution and vigilant monitoring is needed to prevent acute or chronic neurotoxic manifestations.

Currently, no standard therapeutic regimen nor gold standard biomarkers are available to take care of this issue but based on preclinical and clinical studies, various drugs have been repurposed for possible neuroprotective effects. Considering the pharmacotherapeutic approach, methylphenidate, memantine, statins, NSAIDS and a few other drugs have beneficial effects. Acetylcholinesterase inhibitors and VEGF-A inhibitors have also been explored for the possible therapeutic benefit (Peng et al., 2021). However, studies had shown a deleterious effect on the hippocampus when these drugs were used (Pakzad et al., 2014). Studies have also shown that over-expressed PKC in response to radiotherapy causes damage to the hippocampus and causes neuroinflammation and cognitive dysfunction. Therefore, PKC inhibitors, chelerythrine, and midostaurin have been used for possible effects (Makale et al., 2017). Additionally, the use of benzothiazole amphiphiles significantly reversed the neurotoxic manifestations of radiotherapy. Stem cell therapy has also shown a significant neuroprotective effect against bran damage, reduced angiogenesis, and cognitive dysfunction (Fukunaga, 2021).

However, significant clinical outcomes have not been achieved with these drugs and possible reasons could be a multifactorial mechanism of neurotoxicity, pharmacodynamic, and pharmacokinetic limitation. Therefore, various natural and synthetic drugs encapsulated in nanocarriers and miRNA-based therapy have been explored in terms of targeted therapy to overcome the existing limitations (Iqubal et al., 2021).

Thus, we are in consideration with the recommendation that the dose of radiotherapy must be kept minimum. Radiotherapy must be exposed to the tumor region and not the healthy region. More long-term follow studies should be conducted to understand better the mechanism of neurotoxicity and neuroprotective effect of various pharmacotherapy. Moreover, nanocarrier based drug delivery system should be explored for the neuroprotective potency against radiotherapy-induced neurotoxic manifestations. We further suggest that using the natural product and their nanocarrier with well-validated scientific evidence must be promoted and considered for clinical use. Additionally, regulations and policies should be developed for the timely translation of preclinical findings into clinical setup. More and more potent drugs can be brought from bench to bedside to achieve significant improvement in patients’ quality of life.
